# Scurvy: Dietary Discretion in a Developed Country

**DOI:** 10.5811/cpcem.2018.1.36860

**Published:** 2018-03-14

**Authors:** Megan E. Perry, Nathan Page, David E. Manthey, Joshua M. Zavitz

**Affiliations:** * Wake Forest School of Medicine, Winston-Salem, North Carolina; † Wake Forest School of Medicine, Department of Emergency Medicine, Winston-Salem, North Carolina

## Abstract

Although the causes have changed, scurvy (vitamin C deficiency) is still diagnosed in developed countries. We report a case of an 18-year-old female who presented to our emergency department with thrombocytopenia, sinus tachycardia, hypotension, fatigue, gingival hyperplasia, knee effusion, petechiae and ecchymosis in lower extremities. The differential diagnosis included hematologic abnormalities, infectious etiologies, vasculitis and vitamin deficiency. A brief dietary history was performed revealing poor fruit and vegetable intake, thus increasing our suspicion for vitamin C deficiency. This experience illustrates the importance of a dietary history and reminds us to keep scurvy in the differential diagnosis.

## INTRODUCTION

Vitamin C deficiency, commonly known as scurvy, was originally a disease of sailors, related to extended periods of time on a ship with no access to fresh fruit or vegetables.^1^ Dr. Lind of the Royal Navy in 1747 identified citrus fruit as a treatement for scurvy. It is also documented historically during times of famine, where those same sources of vitamin C were inaccessible to the population.^2^ Although the presentation remains the same, the risk factors of developing scurvy today have changed. The rare cases seen in the present day are often observed in populations that frequent the emergency department (ED) and include those with poor nutritional intake related to mental illness, alcohol abuse, isolation and extremely restricted diets.^2^ If untreated, scurvy, although easily remedied, can be fatal. Thus, a dietary history is necessary to identify those at risk for vitamin C deficiency for timely diagnosis.

This case describes a young female who presented to the ED with fatigue, dyspnea, gingival bleeding, scattered ecchymoses, knee swelling and petechiae. A dietary history revealed a limited intake of only specific foods raising a concern for vitamin deficiency. Other diagnostic considerations included coagulopathies, immune-mediated disorders, trauma, vasculitis, liver disease and malignancy. The clinical picture along with a low ascorbic acid (vitamin C) level confirmed the diagnosis of scurvy. This case highlights the importance of a wide differential and a dietary history, which can lead to quick resolution of a potentially fatal disease.

## CASE REPORT

An 18-year-old woman presented to the ED as instructed by her primary care physician for a laboratory abnormality of low platelets with specific concern for idiopathic thrombocytopenic purpura. One month prior to presentation to the ED, the patient noted some shortness of breath on exertion. Three weeks prior to presentation, she developed a rash on her legs, along with bruising, and right knee swelling that made it difficult to ambulate. She also endorsed increased fatigue, weakness, and bleeding from her gingiva. She had been seen several times previously by her primary care physician, but a diagnosis had not yet been discovered. The patient denied any recent trauma, fevers, weight or appetite changes, chest pain, cough, nausea, abdominal pain, constipation, diarrhea, bloody stools, hematuria, or menorrhagia.

Her past medical history was significant for anxiety, depression, asthma and anorexia nervosa (since resolved) and specifically negative for coagulopathies. Her only medications included escitalopram and albuterol inhaler. Family history was noncontributory and specifically negative for coagulopathies, autoimmune disorders and leukemia. Social history was significant for a specific diet consisting of only peanut butter crackers, strawberry Nutri-Grain bars, and fish sticks. She denied tobacco, alcohol or illicit drug use.

**Image 1:**
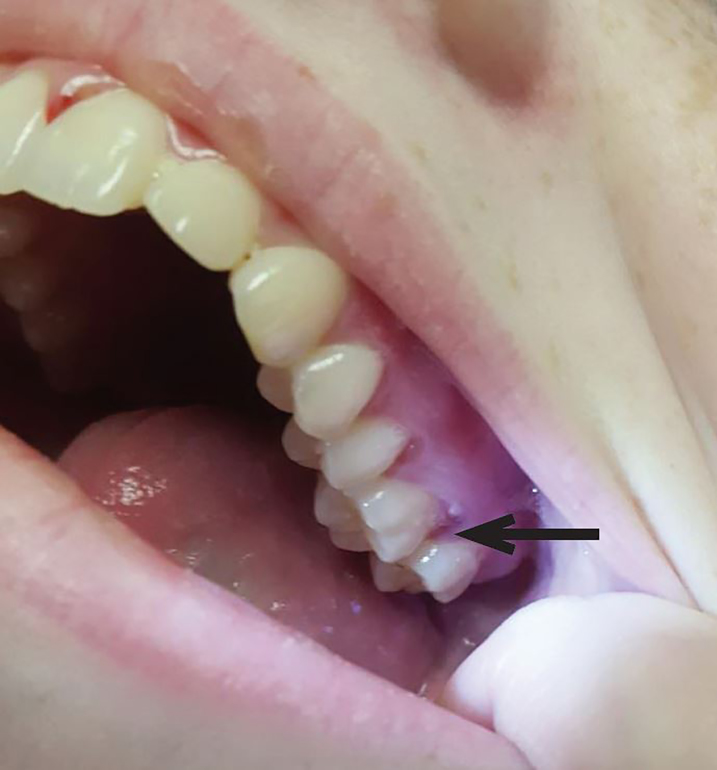
Gingival hyperplasia, most prominent in the patient’s left upper gum line (arrow), is a typical sign of scurvy.

**Image 2:**
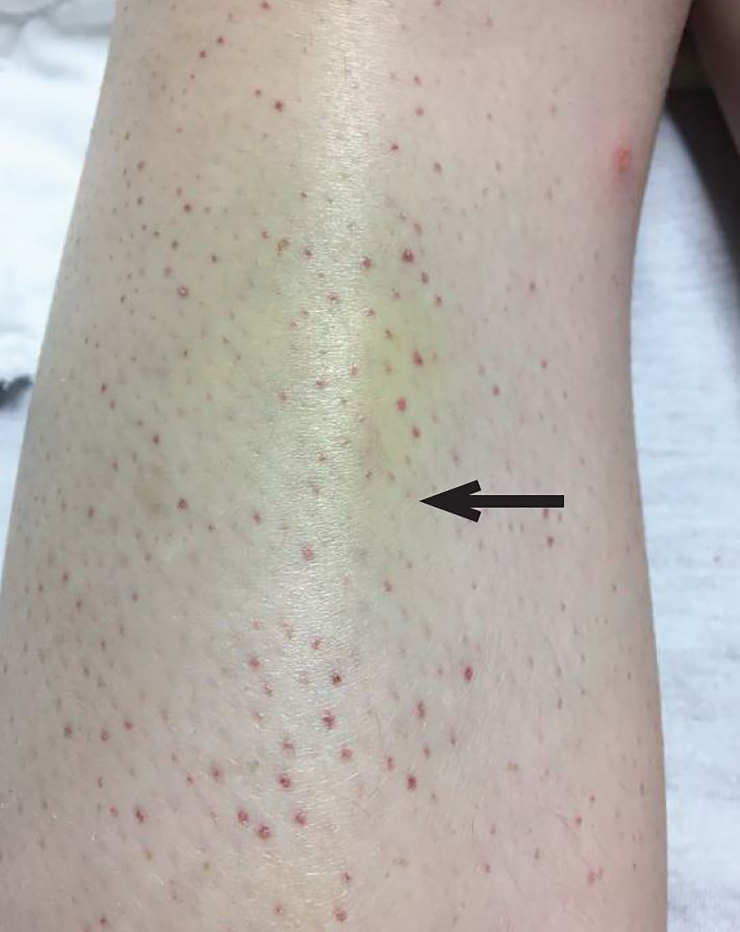
Petechiae, which classically circumscribe the hair follicles, and healing ecchymosis noted on the patient’s bilateral lower extremities (arrow).

**Image 3:**
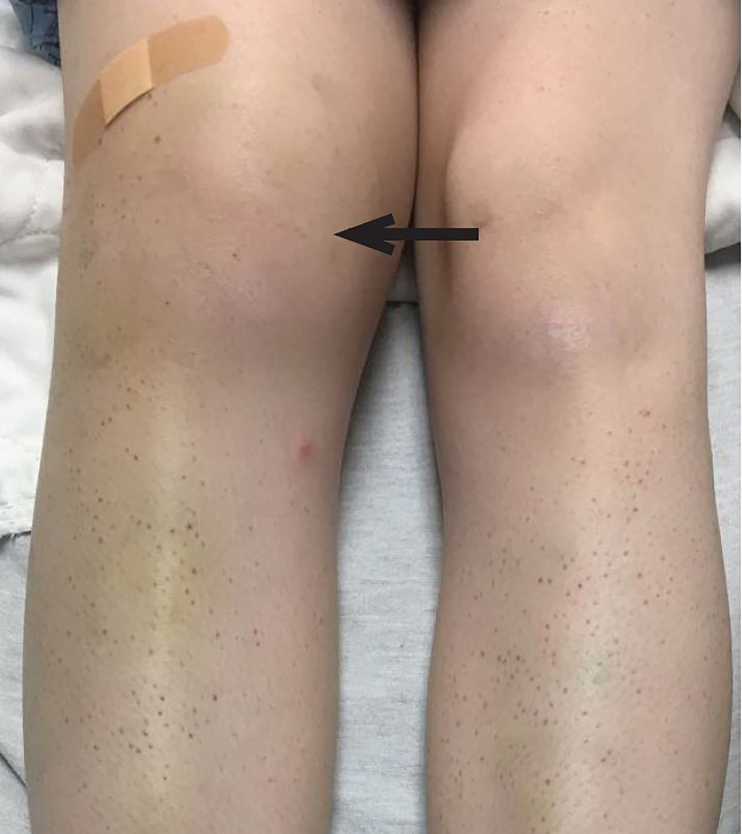
Joint effusion noted in the right knee (arrow), which when explored by arthrocentesis was revealed to be a hemarthrosis.

On physical examination, the patient was afebrile, hypotensive to 86/58 mmHg, tachycardic to 112 beats per minute, with a respiratory rate of 15 breaths per minute and an oxygen saturation of 100% on room air. She was pale and thin appearing, in no acute distress. Oral examination was significant for a region of gingival hyperplasia along the left upper gingival line (Image 1), and petechiae beneath the tongue. Cardiovascular exam was significant for a tachycardic rate, but regular rhythm, 2+ peripheral pulses and normal capillary refill. Pulmonary and abdominal exams were within normal limits. Skin exam demonstrated petechiae on the bilateral lower extremities along with scattered ecchymoses (Image 2). The right knee was also swollen and ecchymotic (Image 3) with tenderness along the joint line and decreased range of motion secondary to pain. The remainder of the physical exam was within normal limits.

ED laboratory results were significant for hemoglobin of 10.5 g/dL, a platelet count of 146 x 10/dL, iron of 32 mcg/dL, C-reactive protein of 34.2 mg/L and negative urine pregnancy test. The remainder of the laboratory studies including complete blood count, coagulation studies, complete metabolic profile, iron profile, urine drug screen, hepatitis panel, and iron profile were all within normal limits. Chest and right knee radiographs were also within normal limits. An arthrocentesis of the right knee joint revealed 600,000 red blood cells and 378 white blood cells. It was negative for crystals and organisms. The patient was admitted to the medicine service for further evaluation with a presumed diagnosis of vitamin C deficiency.

While she was inpatient, laboratory studies demonstrated hematuria, new and worsening anemia and leukopenia, and persistent thrombocytopenia. Anti-nuclear antibody, direct antiglobulin profile, copper and vitamin D were all within normal limits. An ascorbic acid level was <0.1 mg/dL (normal 0.2–1.5 mg/dL). Dermatology believed the patient’s presentation to be consistent with vitamin C deficiency; however, she declined confirmatory biopsy of petechial lesions. The patient received vitamin C supplementation. After improvement of all laboratory values, she was discharged home to continue vitamin C and iron supplementation outpatient. Follow-up with the hematologist demonstrated vitamin C compliance and resolution of all symptoms.

## DISCUSSION

This non-ill appearing patient presented with tachycardia and borderline low blood pressure, fatigue, petechiae and scattered ecchymosis in lower extremities, gingival hyperplasia, and right knee effusion. The differential included the broad categories of hematologic abnormalities, vitamin deficiencies, trauma, vasculitis, liver disease, infection and malignancy.

Hematologic abnormalities included idiopathic thrombocytopenic purpura, thrombotic thrombocytopenic purpura, hemolytic uremic syndrome, disseminated intravascular coagulation, hemophilia (A, B, C), and drug-induced thrombocytopenia. Vitamin deficiencies included vitamin K, zinc, and vitamin C (scurvy). Infectious causes included meningococcemia, Rocky Mountain spotted fever, and septic arthritis. Vasculitis causes included leukocytoclastic vasculitis and Henoch-Schonlein purpura. Idiopathic thrombocytopenic purpura, the original diagnosis of the referring physician, is a disease process of antiplatelet antibodies in which the patient does not appear ill. Although the patient has a decrease in his/her platelet count, all other hematologic parameters are normal. The presentation is one of easy and/or excessive bruising (purpura), petechiae (usually on the lower legs), bleeding from gingiva, blood in urine, and unusually heavy menstrual flow.

**CPC-EM Capsule**

What do we already know about this entity?

Scurvy is a disease of vitamin C deficiency secondary to poor nutritional intake causing anemia, fatigue, spontaneous bleeding, joint swelling, and gingival ulceration.

What makes this presentation of disease reportable?

This case was a rare presentation of scurvy in a young female presenting with petechia, purpura and a joint effusion to an emergency department in a developed country.

What is the major learning point?

Scurvy has significant morbidity and is still diagnosed in developed countries today, usually among patients with poor nutrition such as alcoholics, homeless, and those on fad diets.

How might this improve emergency medicine practice?

This reminds emergency providers of the importance of dietary history and to keep scurvy in the differential diagnosis.

Thrombotic thrombocytopenic purpura is caused by clotting in small blood vessels due to endothelial defect. The disease presents with any variation on the pentad of microangiopathic hemolytic anemia, thrombocytopenic purpura, fever, neurologic abnormalities, and renal disease in an ill-appearing patient. The laboratory data should show anemia with schistocytes, mild increase in fibrin degradation products and possible increase in prothrombin time (PT) and partial thromboplastin time (PTT).

Disseminated intravascular coagulation (DIC) is a diverse entity due to thrombin excess caused by systemic activation of blood coagulation that presents with clotting disorders. Accelerated fibrinolysis due to the clot formation may also actually cause severe bleeding. The patient will present ill appearing, most commonly from the underlying disorder that triggered the DIC. DIC will cause thrombosis, embolism, organ dysfunction (due to clotting) and bleeding. This severe consumptive coagulopathy will present with anemia, thrombocytopenia, elevated fibrin degradation factors, and elevated PT/PTT.

Leukemia can present in a multitude of ways, depending on the type of leukemia, but common symptoms include fatigue, loss of weight, swollen lymph nodes, easy bleeding/bruising, petechiae, and bone pain. Hemophilia presents as bruising, excessive bleeding after cuts, pain and swelling in joints, blood in stool. This would be unusual as the patient was female, had no prior bleeding issues, and had no family history. Women with hemophilia are most commonly asymptomatic simple carriers. Hemophilia C occurs in both sexes, with mild symptoms, caused by insufficient clotting factor XI. Vasculitis (microangiopathic, leukocytoclastic, drug induced) often presents with fatigue, fever, arthralgias and weight loss. The patient may present with sensory or motor deficits, vascular abnormalities, palpable purpura or petechiae. The diagnosis is based on a combination of involved organ systems, the size of vessels affected, and characteristics of testing results.

Scurvy (vitamin C deficiency) presents with anemia, fatigue, spontaneous bleeding (bruising and petechiae), joint swelling (hemarthrosis), ulceration of the gingiva with gingival hyperplasia, and eventual loss of teeth. Modern cases of vitamin C deficiency are rare but usually found in alcoholic patients, isolated elderly patients, malabsorption syndromes, and people who voluntarily restrict their type of food intake. In addition to poor nutritional intake, alcoholic patients develop vitamin C deficiency secondary to increased excretion of vitamin C in the urine.^3^ The recommended daily allowance of vitamin C is 60 mg/day. Scurvy develops in approximately four weeks in those who consume less than 10 mg/day. Daily requirements increase for patients who are pregnant (70 mg/day) and during lactation (90–95 mg/day), or patients who smoke, are on hemodialysis, or have trauma/infection.^2^

We rely on exogenous vitamin C from various foods in our diet, such as fresh fruits and vegetables (oranges, lemons, limes, potatoes, broccoli, spinach, red peppers, etc.). Fresh meat contains vitamin C; however, it is destroyed when the meat is cooked.^4^ Vitamin C is a vital required cofactor for collagen biosynthesis and plays an important role in carnitine biosynthesis, production of noradrenaline, metabolism of cholesterol, iron absorption, antioxidant activity, corticosteroid synthesis, and various drug-metabolizing systems. Most symptoms of vitamin C deficiency can be attributed to impaired formation of mature connective tissue, such as bleeding in the skin (ecchymosis, perifollicular hemorrhages, petechiae), joints, pericardium, adrenal glands, and peritoneal cavity, as well as inflamed and bleeding gingiva.^5^ In children, bone growth is impaired with a deficiency of vitamin C and can be associated with bleeding into the periosteum and sub-periosteum.^6^

Scurvy progresses in four stages. The first stage is characterized by muscle pain and fatigue. The second stage shows gingival swelling with associated easy bleeding. The third stage reveals ulcerative gingivitis, non-palpable purpura and petechiae, and ulcers. The fourth stage is identified by multiple organ failure.^2^ The treatment of vitamin C deficiency is simply supplementation of vitamin C. While a standardized treatment protocol is not established, multiple sources recommend one g/day of vitamin C for the first two to three days followed by 500 mg/d for the next week.^2^,^7^ It is then suggested that 100 mg/d should be taken for one to three months. Systemic symptoms (fatigue, pain, anorexia, confusion) should improve within 24 hours of treatment. Bleeding and associated collagen defects (ecchymosis, petechiae, gingival bleeding and hyperplasia, perifollicular hemorrhage) should begin improving within one to two weeks. One should anticipate approximately three months until complete recovery with regular vitamin C supplementation.^7^

## CONCLUSION

Scurvy is often taught as a historical disease and is rare in modern society. In this case, a primary care physician had left it off the differential diagnosis. Presentation of patients with petechiae and purpura is not uncommon in the emergency department and we need to keep scurvy on the differential. This case also highlights the importance of taking detailed histories (in this case of diet) to help increase or decrease the likelihood of a disease.

Documented patient informed consent and/or Institutional Review Board approval has been obtained and filed for publication of this case report.
